# A novel complementary pathway of cordycepin biosynthesis in *Cordyceps militaris*

**DOI:** 10.1007/s10123-023-00448-9

**Published:** 2023-11-21

**Authors:** Hucheng Zhang, Jun Yang, Shuai Luo, Linying Liu, Guowei Yang, Bo Gao, Haitao Fan, Lina Deng, Ming Yang

**Affiliations:** 1 Bioengineering College Beijing Polytechnic, Beijing, 100176 China; 2 Department of English, Beijing Health Vocational College, Beijing, 102402 China; 3 Department of Cardiovascular Surgery Institute of Cardiac Surgery, PLA General Hospital, Beijing, 100141 China

**Keywords:** *Cordyceps militaris*, Cordycepin, Transcriptome analysis, Biosynthetic pathway, Adenosine

## Abstract

We determined whether there exists a complementary pathway of cordycepin biosynthesis in wild-type *Cordyceps militaris*, high-cordycepin-producing strain *C. militaris* GYS60, and low-cordycepin-producing strain *C. militaris* GYS80. Differentially expressed genes were identified from the transcriptomes of the three strains. Compared with *C. militaris*, in GYS60 and GYS80, we identified 145 and 470 upregulated and 96 and 594 downregulated genes. Compared with GYS80, in GYS60, we identified 306 upregulated and 207 downregulated genes. Gene Ontology analysis revealed that upregulated genes were mostly involved in detoxification, antioxidant, and molecular transducer in GYS60. By Clusters of Orthologous Groups of Proteins and Kyoto Encyclopedia of Genes and Genomes analyses, eight genes were significantly upregulated: five genes related to purine metabolism, one to ATP production, one to secondary metabolite transport, and one to RNA degradation. In GYS60, cordycepin was significantly increased by upregulation of ATP production, which promoted 3′,5′-cyclic AMP production. Cyclic AMP accelerated 3′-AMP accumulation, and cordycepin continued to be synthesized and exported. We verified the novel complementary pathway by adding the precursor adenosine and analyzing the expression of four key genes involved in the main pathway of cordycepin biosynthesis. Adenosine addition increased cordycepin production by 51.2% and 10.1%, respectively, in *C. militaris* and GYS60. Four genes in the main pathway in GYS60 were not upregulated.

## Introduction

*Cordyceps militaris*, a model parasitic fungus species of the family Cordycipitaceae and genus *Cordyceps*, has been a traditional Chinese medicine for centuries (Zhu et al. 1998). In Asia, because of a wide range of biological effects, including immune regulation, antitumor, antioxidation, anti-inflammatory, and antimicrobial properties (Tuli et al. 2014; Zhang et al. 2019; Qin et al. 2019; Jędrejko et al. 2021; Kontogiannatos et al. 2021), *C. militaris* has been used as an exhaustion remedy and treatment for numerous diseases. In 2009, the Ministry of Health, People’s Republic of China, officially proclaimed the fruiting body of *C. militaris* to be edible (Ministry of Health of the People’s Republic of China 2009), which promoted further research of the fungus. *Cordyceps militaryis* contains many bioactive substances, such as cordycepin, adenosine, cordyceps polysaccharide, amino acids, ergosterol, and polycystin (Zhu et al. 1998; Jędrejko et al. 2021; Quy et al. 2019). Among these substances, cordycepin is the most important and the standard by which to measure the quality of *C. militaris* (Qin et al. 2019).

Cordycepin, 3′-deoxyadenosine, is the first adenosine analog isolated from *C. militaris* (Cunningham et al. 1951). Cordycepin has antitumor (Cui et al. 2018; Xu et al. 2019; Khan and Tania 2020; Zheng et al. 2020), antibacterial (Jiang et al. 2019), antifungal (Sugar and Mccaffrey 1998), antivirus (Verma 2020), antioxidation (Olatunji et al. 2016), and immune regulation properties (Wang et al. 2020); thus, cordycepin has important value in commerce, medicine, and scientific research (Qin et al. 2019). Recently, the yield of cordycepin was improved from 380 to 6840 mg/L by chemical synthesis (Huang et al. 2018), optimization of liquid fermentation (Lee et al. 2019), extraction from fruit bodies (Wen et al. 2016), and genetics (Das et al. 2010). Previously, we used the multifunctional plasma mutagenesis system to create a high-cordycepin-producing strain, named *C. militaris* GYS60, whose yield of cordycepin was 7883 mg/L. We also created a low-cordycepin-producing strain GYS80 (Zhang et al. 2020).

**Fig. 1:**
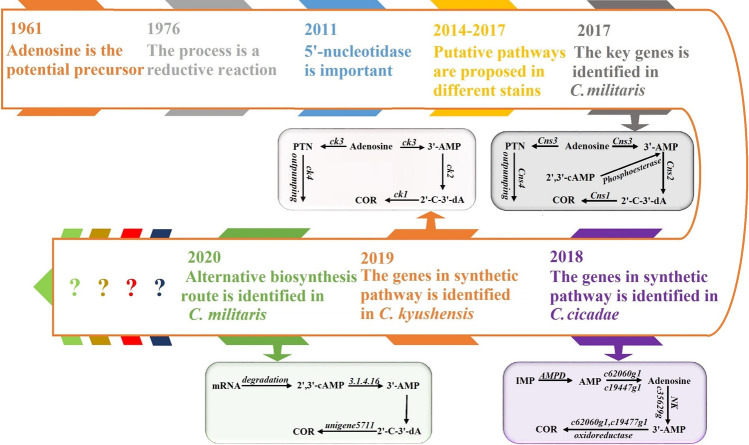
Research history and schematic representation of cordycepin biosynthesis pathway (Yang et al. 2020). Note: COR, cordycepin; 3′-AMP, adenosine-3′-monophosphate; IMP, inosine monophosphate; 2′-C-3′-dA, 2′-carbonyl-3′-deoxyadenosine; PTN, pentostatin; 2′,3′-AMP, adenosine 2′,3′-cyclic monophosphate; AMPD, adenosine monophosphate deaminase; mRNA, messenger ribonucleic acid

The cordycepin biosynthetic pathway has been studied to further improve the yield. Figure 1 shows the research history of cordycepin biosynthesis. Adenosine, as a precursor (Kredich and Guarino 1961), is reduced to cordycepin (Lennon and Suhadolnik 1976). In 2011, a 5′-nuclease was found as an important key enzyme in the biosynthetic pathway of cordycepin (Zheng et al. 2011), which, with adenosine kinase and adenylate kinase, participates in conversion of 3′-dAMP to cordycepin (Xiang et al. 2014). In 2017, four other enzymes with key functions in cordycepin biosynthesis (Xia et al. 2017; Liu et al. 2018; Raethong et al. 2018; Zhao et al. 2019) were discovered and registered in the National Center for Biotechnology Information (NCBI). These four enzymes are ATP-binding cassette transporters, nucleotide kinase, phosphohydrolase, and oxidoreductase. In 2019, 2′-carbonyl-3′-deoxyadenosine (2′-C-3′-dA) was found as a precursor for the production of adenosine 3′-phosphate (Wongsa et al. 2020).

So far, the main pathway of cordycepin biosynthesis is clear. Adenosine is phosphorylated to 3′-AMP by nucleotide kinase (Cns3), then dephosphorylated to 2′-C-3′-dA by phosphohydrolase (Cns2), and cordycepin is finally produced by oxidoreductase (Cns1). Accordingly, the four genes involved with cordycepin synthesis were cloned in *Saccharomyces cerevisiae* to produce 137.27 mg/L (Huo et al. 2021) cordycepin, a yield that was lower than the 380 mg/L produced by *C. militaris*. The *S. cerevisiae* study indicated that there are other complementary pathways for cordycepin synthesis in *C. militaris*.

On the basis of our previous experiments (Zhang et al. 2020), we analyzed gene expression differences between the three strains GYS60, GYS80, and *C. militaris* to investigate whether there is a complementary pathway of cordycepin biosynthesis. The existence of a complementary pathway was confirmed by the effect on cordycepin biosynthesis of adding adenosine during fermentation and comparing the expression of the four key genes indicated earlier.

## Materials and methods

### Materials

High-performance liquid chromatography was performed with an Agilent 1200 system (Agilent Technologies, Santa Clara, CA, USA) and a Cosmosil C18-AR-II, 5 µm, 4.6 mm × 250 mm column (Nacalai Tesque, Kyoto, Japan). A NanoDrop Microvolume Spectrophotometer 2000 and Fluorometer Qubit 4.0 were obtained from Thermo Fisher Scientific (USA). A HE-120 electrophoresis and gel imaging analysis system were from Tanon-2500 (Tianneng Technology Co., Ltd, China). A Centrifuge5 418R was from Eppendorf (Germany).

Authentic cordycepin was purchased from Sigma-Aldrich (USA). Chromatographically pure methanol was from Merck (Darmstadt, Germany). RNA Nano 6000 kits were obtained from Agilent, and mRNA extraction kit DP411 was from Tiangen Biochemical Technology Co., Ltd (Beijing, China). VAHTS Universal V6 RNA-seq Library Prep Kit NR604-02 for Illumina® was from Nanjing Vazyme Biotech Co., Ltd, China. Other reagents were purchased from Sinopharm Chemical Reagent Co., Ltd, China.

*C. militaris* was purchased in the market. Strains GYS60 and GYS80 were created with a multifunctional plasma mutagenesis system, and the strains were cultivated and stored in our laboratory (Zhang et al. 2020).

### Methods

#### Fermentation of *C. militaris* and adenosine addition

*C. militaris*, GYS60, and GYS80 were inoculated on PDA medium and cultured at 28 ℃ for 7 to 14 days. When the mycelium almost filled the entire Petri dish, several mycelium agar plugs were cut out with a 1.0-cm-diameter cork borer, and six plugs were placed into 250-mL fermentation vessels containing 50 mL seed culture medium (potato 200.0 g/L, glucose 40.0 g/L, acid-hydrolyzed casein 4.0 g/L, peptone 3.0 g/L, yeast extract 3.0 g/L, KH_2_PO_4_ 2.0 g/L, NH_4_NO_3_ 5.0 g/L, MgSO_4_ 0.2 g/L), and shaken at 28 ℃ for 5 days at 150 r/min. Seeds (10%) were put into a 500-mL flask with 200 mL of fermentation liquid culture medium and cultured under the same conditions. Each strain was cultured three times. After 15 days, the cells were collected for transcriptome sequencing and HPLC analysis of adenosine and cordycepin in the supernatant.

To investigate the influence of adenosine on cordycepin synthesis, each strain was tested three times. On the seventh day, adenosine at a final concentration of 1.0 mg/mL was added to the fermentation liquid, and the culture was continued until the 20th day. From the first day, 2.0 mL fermentation liquid was taken every 2 days to measure the dry weight of the cells and for HPLC analysis of adenosine and cordycepin in the fermentation medium.

#### Measurement of adenosine and cordycepin by HPLC

Analysis, preparation of a standard curve, and content calculation were performed as described (Zhang et al. 2020). The mobile phase was water (A) and methanol (B), and gradient elution was used. The elution procedure was 0–5 min, 5% B; 5–10 min, 5–15% B; 10–20 min, 15% B; 20–25 min, 15–100% B; and 25–30 min, 100% B. The flow rate was 1.0 mL/min, and the sample volume was 10 μL. The detection wavelength was 260 nm.

#### RNA extraction, sequencing, de novo assembly, and analysis

The extraction, sequencing, and assembly of total RNA from *C. militaris*, GYS60, and GYS80 were completed by Beijing Biomarker Biotechnology Co., Ltd, China. Total RNA was extracted according to the instructions of the DP411 kit. RNA concentration and purity were analyzed with a micro UV spectrophotometer (Nano Drop 2000) and a Qubit 4.0 Fluorometer. RNA integrity and genome contamination were assessed with an Agilent biological analyzer 2100 and RNA Nano 6000 kit. The qualified RNA was used for sequencing. An Illumina Novaseq 6000 was used to build a sequencing library and analyze three samples in parallel to prevent error.

The 3′ end sequencing connector was removed from the raw sequencing data, sequences less than 20 bases were removed, and the unknown base sequences in reads were removed to obtain clean reads data. Hisat2 software was used to extend the sequence into a contig by overlapping sequences of clean reads, and then, the contig was connected into a transcript according to the paired end sequencing information. The splicing sequence was deduplicated, and the sequence with a length greater than 200 bp was taken. From the assembled sequences, the longest transcripts from potential alternative splicing were selected as the independent gene sequence of the sample. Finally, the simple sequence repeats of the uniform sequences were analyzed and compared with the Gene Ontology, Clusters of Orthologous Groups of Proteins, and the Kyoto Encyclopedia of Genes and Genomes (KEGG) databases. A similarity greater than 30% and e less than 10^−5^ were taken to get the comment information of unigenes. Transcriptome information was analyzed by BMKCloud (www.biocloud.net).

#### Differential expression analysis of four key genes in principal cordycepin synthesis pathway

Annotation information and gene expression levels of the four key genes cordycepin synthesis genes *Cns*1, *Cns*2, *Cns*3, and *Cns*4 were searched in BMKCloud (www.biocloud.net), and differential expression analysis was conducted.

#### Quantitative real-time PCR validation of differentially expressed genes

The *C. militaris* strains were cultured as described to the 15th day of the late logarithmic phase, then centrifuged at 10,000 × g at 4 ℃ for 5 min. Total mRNA was extracted from the mycelia according to the instructions of the mRNA extraction kit DP411. The concentration and purity of the mRNA were measured with the NanoDrop spectrophotometer and Fluorometer Qubit 4.0. The mRNA was reverse transcribed into cDNA and stored at 4 ℃. All primers were designed at https://www.ncbi.nlm.nih.gov/tools/primer-blast/index.cgi?LINK_LOC=BlastHome (see Table 1). The RT-qPCR reactions were 20 µL, containing 50 ng of cDNA, 160 nmol/L primers, and the SYBR Green Super Real PreMix Plus (Tiangen, Beijing, China). All qRT-PCR reactions were conducted in the CFX96 Real-Time PCR Detection System (Bio-Rad, CA, USA), according to the reaction parameters provided. The 18S rDNA was used as an internal control. The relative gene expression level was calculated by the 2^−△△CT^ method using the formula △△CT = (CT_target_–CT_rDNA_) _interested sample_–(CT_target_–CT_rDNA_) _calibrated sample_. We selected the expression of each gene on the first day of inoculation as the calibrated sample, and the 15th day as the interest sample.

**Table 1:** Primers for RT-qPCR

Name	Forward primers (5′ → 3′)	Reverse primers (5′ → 3′)
18S rDNA	AGGTTTCGGGAATGTGGCTC	AGAACATCAGGATCGGTCGG
CCM_07507 (oxidoreductase)	GAAGTCACACGATGGGAGCA	AGGCACGGAGGTAGTAGAGG
CCM_02777 (3′,5′-cyclic-AMP phosphodiesterase)	GCACAAACCTGTGCCGTTTA	GTGACGTTGGATTCTTGCGG
CCM_04722 (transferase)	GATGCTTCGATGTTGGCGAC	AGCATGCAACGAACAACACC
CCM_06928 (adenylate cyclase)	GCGGTACCTCAACCTATCCG	CGCTTTGATCGGGAATGCTG
CCM_04436 (oxidoreductase/dehydrogenase)	GCTCACGAGTTCGTCACTCA	TCGAAGCTCGTGCTCATTGT
CCM_04437 (metal-dependent phosphohydrolase)	CATTTCCGAGTGCGACAACG	CACCTTGTCCTCGACAGAGC
CCM_04438 (nucleotide kinase)	CGAGACGGTCTACAAGGAGC	GTTGTAGACGTGGTTCGGGT
CCM_04439 (ABC transporter)	AACTATCCAAACTCGCCGCA	GCCACGGTGCTTTGCTAAAA

#### Statistical analysis

Origin 8.5 software was used for mapping and statistics; *p* < 0.05 was considered to represent significance.

## Results

### Mycelia mass and cordycepin production

The three strains showed similar growth profiles and maximum growth rate (*µ*_max_) in liquid fermentation medium (Table 2). The maximum biomass was reached at the 15th day and remained unchanged thereafter. The three strains began to produce cordycepin on the tenth day; the maximum value was reached on the 15th day, and remained stable until decreasing on the 19th day. These results were consistent with other reports (Suparmin et al. 2017).

**Table 2:** Growth characteristics of the three strains (*n* = *3*, *¯x* ± *SD*)

Growth characteristics	*C. militaris*	*C. militaris* GYS60	*C. militaris* GYS80
Maximum specific growth rates (*μ*_max_)	0.200 ± 0.010	0.210 ± 0.020	0.190 ± 0.030
Biomass productivity (g/L/day)	3.420 ± 0.030	3.510 ± 0.050	3.340 ± 0.040
Extracellular total cordycepin titer (g/L)	0.381 ± 0.010	7.883 ± 0.108	0.154 ± 0.016
Extracellular cordycepin productivity (g/L/day)	0.025 ± 0.001	0.526 ± 0.003	0.011 ± 0.001

The growth state of three *C. militaris* strains was approximately the same, and the hyphae were taken at the end of the log phase (15th day) for transcriptome analysis and adenosine and cordycepin content analysis

### Gene function and differential expression analysis

To determine whether *C. militaris* contains a complementary pathway of cordycepin biosynthesis, we compared gene expression levels of *C. militaris*, GYS60, and GYS80. Total mRNA of these strains was sequenced with three biological replicates. Two hundred twenty-eight Mb of total clean reads was obtained (Supplementary Table S1). The total clean bases of each sample was greater than 5.98 Gb (Supplementary Table S1). We compared the clean reads of each sample with the cordyceps reference genome (www.ncbi.nlm.nih.gov/genome/?Term=cordyceps+militaris) (Supplementary Table S2).

**Fig. 2:**
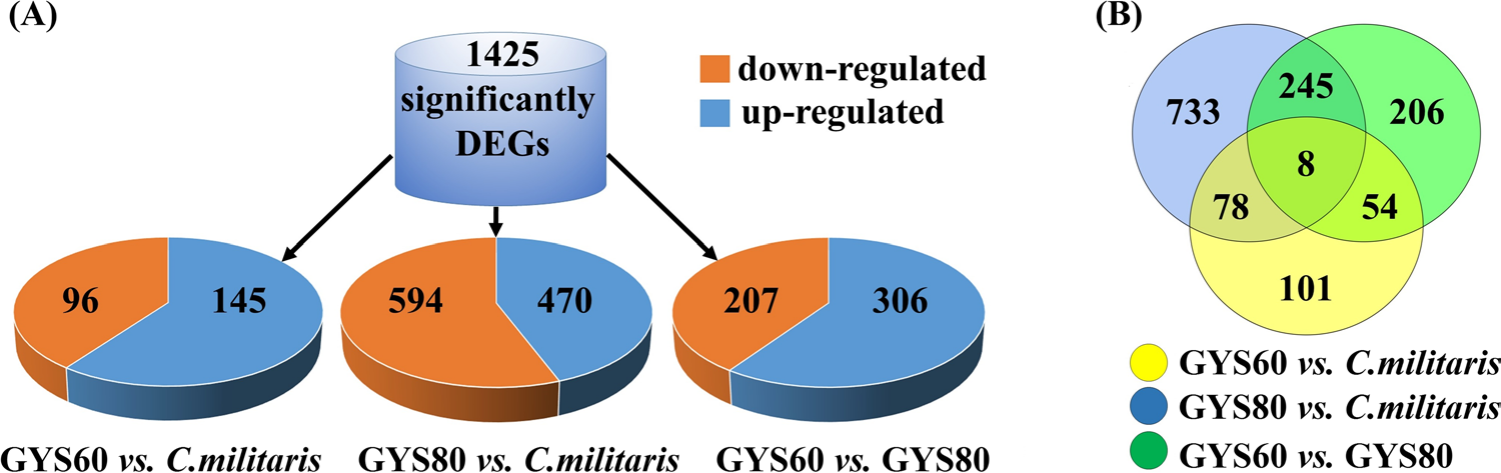
Gene expression profiles of GYS60, *C. militaris*, and GYS80. **A** The numbers of up- and downregulated genes are shown. **B** Venn diagram of the number of differentially expressed genes based on three comparisons of GYS60 *vs. C. militaris*, GYS80 *vs. C. militaris*, and GYS60 *vs.* GYS80. Note: *C. militaris*, wild type; GYS60, high-cordycepin-producing strain; GYS80 low- cordycepin-producing strain

According to the comparison results, prediction of alternative splicing, and optimization of gene structure, we identified new genes. There were 11,214 genes, 1117 unigenes, and 1425 important genes (Fig. 2A, Supplementary Table S3), of which 302 genes were annotated. There were eight genes with significantly different expression between *C. militaris*, GYS60, and GYS80 (Fig. 2B). When compared with *C. militaris*, we found 145 genes that were upregulated and 96 genes were downregulated in GYS60 (Supplementary Table S4). When compared with *C. militaris*, we found 470 genes were upregulated and 594 genes were downregulated in GYS80 (Supplementary Table S4). Compared with GYS80, GYS60 had 306 upregulated genes and 207 downregulated genes (Fig. 2A, Supplementary Table S4).

**Fig. 3:**
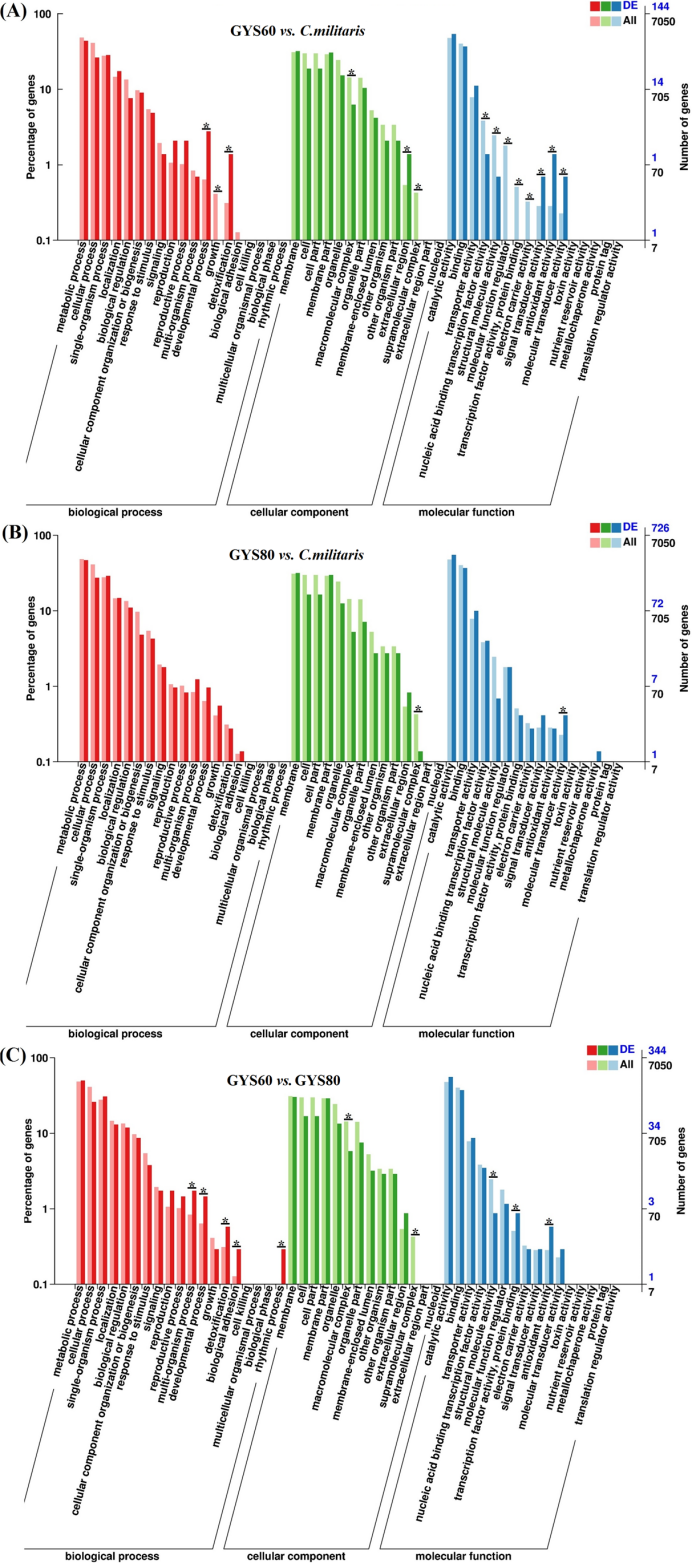
Gene Ontology analysis of transcriptome in *C. militaris* strains. Asterisk (*) indicates significance at *P* < 0.01. **A** GYS60 *vs. C. militaris*. **B** GYS80 *vs. C. militaris*. **C** GYS60 *vs.* GYS80

To further characterize the differential expression of genes, we used the Gene Ontology database to analyze the 11,214 genes with a false discovery rate < 0.05 and the fold change (FC) of gene expression pair value log_2_ (FC) > 1 or log_2_ (FC) <  − 1 (Fig. 3). There were 4045 molecular functional genes, 2010 cell component genes, and 996 biological process genes (Supplementary Table S5); another 4163 genes were not annotated.

When we compared GYS60 with *C. militaris*, genes related to detoxification, antioxidant activity, and molecular transducer activity were significantly upregulated, whereas genes related to growth, supramolecular complex, structural molecular activity, transcription factor activity, and electron carriers were significantly down regulated (Fig. 3A). When we compared GYS80 with *C. militaris*, genes related to molecular transducer activity were significantly upregulated, and genes related to structural molecular activity were significantly downregulated (Fig. 3B). A comparison of GYS60 with GYS80 showed that rhythmic process, biological adhesion, development process, detoxification reaction, and antioxidant activity related genes of GYS80 were significantly upregulated, whereas genes related to supramolecular complexes were significantly downregulated (Fig. 3C).

After *C. militaris* was mutated with a multifunctional plasma mutagenesis system (Zhang et al. 2020), we found that metabolic regulation changed considerably. In GYS60, due to the upregulation of genes such as detoxification reaction, antioxidant activity, and molecular transduction activity, cordycepin could not be degraded easily after synthesis. It was rapidly secreted, so the cordycepin content in the cell remained at a low concentration. Cordycepin synthesis was continuously induced, which improved yield.

### Complementary pathway of cordycepin biosynthesis

Adenine, adenosine, AMP, ADP, and ATP are precursors of cordycepin (Kredich and Guarino 1961; Yin et al. 2012; Pang et al. 2018). Thirty-one genes, 21 enzymes, and 25 biochemical reactions are involved in the pathway of cordycepin synthesis that begins with adenine (Vongsangnak et al. 2017). Thus, we analyzed expression of genes related to purine nucleotides in *C. militaris*, GYS60, and GYS80.

Compared with *C. militaris*, we found eight genes that were significantly upregulated in GYS60 (Table 3). When we compared GYS60 with *C. militaris* and GYS80, a new Gene_1809 related to RNA processing and splicing was found, and its differential expression log_2_ (FC) was significantly upregulated, reaching 2.642399367 and 1.850781774, respectively, to increase cordycepin production. Compared with *C. militaris*, we found that the differential expression amount log_2_ (FC) of the gene was only 0.3071911825 in GYS80.

**Table 3:** Differentially expressed genes involved in cordycepin biosynthetic routes

Gene number	Gene ontology annotation	Enzyme committee number	Log_2_ (fold change)		
			GYS60 *vs. C. militaris*	GYS80 *vs. C. militaris*	GYS60 *vs.* GYS80
newGene_1809	RNA processing (Wongsa et al. 2020)		2.642399367↑	0.3071911825	1.850781774↑
CCM_07507	Putative oxidoreductase activity, energy production, and conversion (this study)		2.364553152↑	0.524825957↓	1.788695182↑
CCM_06643	Sulfate adenylyltransferase (this study)	EC:2.7.7.4	2.225284258↑	1.371203335	− 0.222196551↓
CCM_02777	3′,5′-Cyclic-AMP phosphodiesterase activity (this study)	EC:3.1.4.17	2.018750703↑	0.286702204	− 0.512240783
CCM_04722	Transferase activity (this study)		1.977596036↑	0.601331121	1.781970007↑
CCM_06928	Adenylate cyclase (this study)	EC:4.6.1.1	1.903236863↑	0.570408999	− 1.218688129↓
CCM_02755	ATP adenylyltransferase (this study)	EC:2.7.7.53	1.849150089↑	0.628005942	− 1.005299582↓
CCM_03411	Bis(5′-nucleosyl)-tetraphosphatase (this study)	EC:3.6.1.41	1.720213381↑	0.747801367	− 0.821032803
CCM_04439 (*Cns*4)	ATP-binding cassette (ABC) transporters (Xia et al. 2017)		0.215096615	− 0.326585369↓	0.507654644
CCM_04438 (*Cns*3)	Phosphoribosyltransferases (Xia et al. 2017)		0.131031713	− 1.124416139↓	1.230755168↑
CCM_04437 (*Cns*2)	Phosphoribosyl-aminoimidazole-succinocarboxamide synthase (Xia et al. 2017)	EC:6.3.2.6	− 0.211378697	− 3.007175411↓	2.724424295↑
CCM_01353	Adenylate kinase (Yin et al. 2012)	EC:2.7.4.3	− 0.276398468	− 1.212896673↓	0.885450869
CCM_00622	5′-nucleotidase (Liu et al. 2018)	EC:3.1.3.5	− 0.848205518	− 2.625404167↓	1.582100758↑
CCM_04436 (*Cns*1)	Oxidoreductase activity (Xia et al. 2017)		− 0.958621434	− 2.766218086↓	1.839484561↑
CCM_07683	Phosphodiesterase (Wongsa et al. 2020)	EC:3.1.4.16	− 0.352948935	-0.333839099	− 0.040100804

*C. militaris* is wild type. GYS60 is high-cordycepin-producing; GYS80 produced low levels of cordycepin. The selected data were taken from GYS60 with upregulation of log_2_ (fold change) ≥ 1.0 or downregulation of log_2_ (fold change) ≤  − 1 and false discovery rate ≤ 0.05 when compared with *C. militaris* or GYS80. ↑ means upregulation and ↓ means downregulation

The foregoing result was related to a complementary biosynthetic pathway of cordycepin (Fig. 1) (Wongsa et al. 2020). In the complementary pathway of RNA degradation to cordycepin (Fig. 1) (Wongsa et al. 2020) in GYS60, the expression of new Gene_1809 was upregulated, which increased cordycepin production. In this pathway (Wongsa et al. 2020), RNA is degraded to 2′,3′-cyclic-AMP, which is converted into 3′-AMP by 2′,3′-cyclonucleotide-2′-phosphoriesterase encoded by CCM_07683. Finally, 3′-AMP enters the last two steps of the main pathway of cordycepin biosynthesis. However, the differential expression log_2_ (FC) of CCM_07683 in GYS60 was only − 0.352948935 (Table 3), which indicated that cordycepin synthesis by this complementary pathway of RNA degradation in GYS60 was not a primary pathway.

In the principal pathway of cordycepin biosynthesis (Xia et al. 2017), compared with *C. militaris*, the *Cns*1, *Cns*2, *Cns*3, and *Cns*4 genes were not upregulated in GYS60 (Table 3). In GYS80, these genes were significantly downregulated (Table 3). Compared with GYS80, these genes were significantly upregulated in GYS60 (Table 3). These results supported the hypothesis that there exists other biosynthetic pathways in the cordycepin-producing GYS60 strain.

**Fig. 4:**
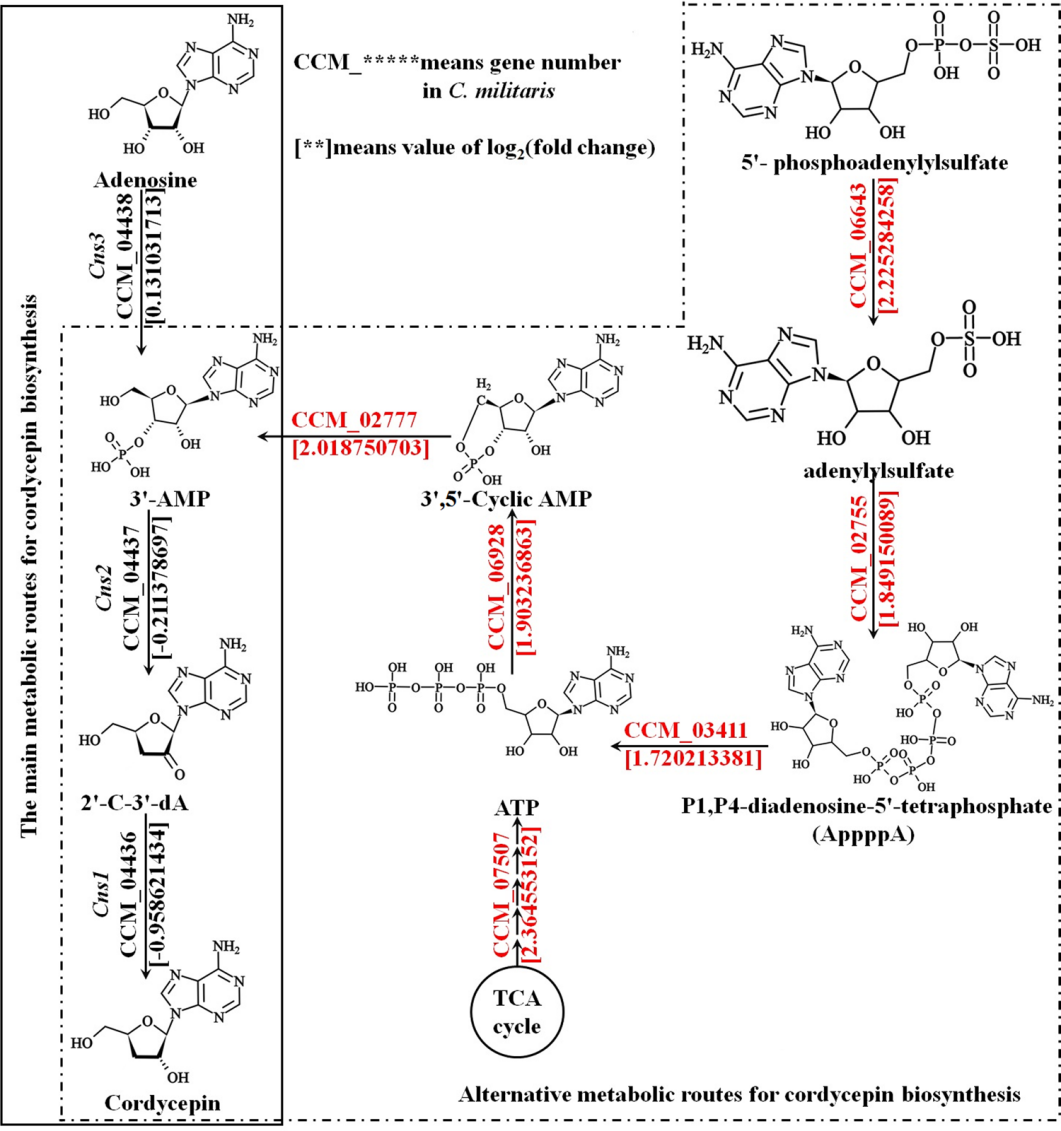
A putative metabolic pathway for 3′-AMP formation in relation to cordycepin biosynthesis in GYS60 compared with *C. militaris.* CCM_ indicates the gene number of *C. militaris* registered in NCBI. Numbers in parentheses indicate the upregulated or downregulated Log_2_ (FC) value. The solid box indicates the main pathway for cordycepin biosynthesis. The dotted box indicates an alternative metabolic pathway for cordycepin biosynthesis. TCA cycle, tricarboxylic acid cycle; ATP, adenosine triphosphate; 3′,5′-AMP, adenosine-3′,5′-cyclic monophosphate; 3′-AMP, adenosine-3′-monophosphate; 2′-C-3′-dA, 2′-carbonyl-3′-deoxyadenosine

Compared with *C. militaris*, we found that six genes in GYS60 were significantly upregulated (Table 3), namely, adenosine sulfate adenylyltransferase, 3′,5′-cyclic-AMP phosphodiesterase, transferase, adenylate cyclase, ATP adenylyltransferase, and bis(5′-nucleosyl)-tetraphosphatase. According to the KEGG metabolic map, adenosyl-phosphate sulfate is catalyzed by adenosyl-sulfate acyltransferase to generate adenosyl sulfate, which is catalyzed by ATP adenosyltransferase to generate P1,P4-diadenosine-5′-tetraphosphate. Then, P1,P4-diadenosine-5′-tetraphosphate is catalyzed by two 5′-nucleoside-tetraphosphatases to generate ATP, which is converted into 3′,5′-cyclic-AMP by adenylate cyclase to generate 3′,5′-cyclic-AMP (Fig. 4). Finally, 3′,5′-cyclic-AMP transformed into 3′-AMP by phosphodiesterase to transform 3′,5′-cyclic-AMP into 3′-AMP, which enters the last two steps of the main pathway of cordycepin biosynthesis.

The increase of cordycepin production by GYS60 was achieved by upregulation of the ATP production rate, increasing the production of 3′,5′-cyclic AMP, accelerating the accumulation of 3′-AMP, and continuing synthesis of cordycepin in the main pathway. Subsequently, these steps lead to secretion of cordycepin by two transporters to enable continuous synthesis of cordycepin. Ultimately, the cordycepin production of the strain was significantly increased.

### Verification of complementary pathway of cordycepin biosynthesis

**Fig. 5:**
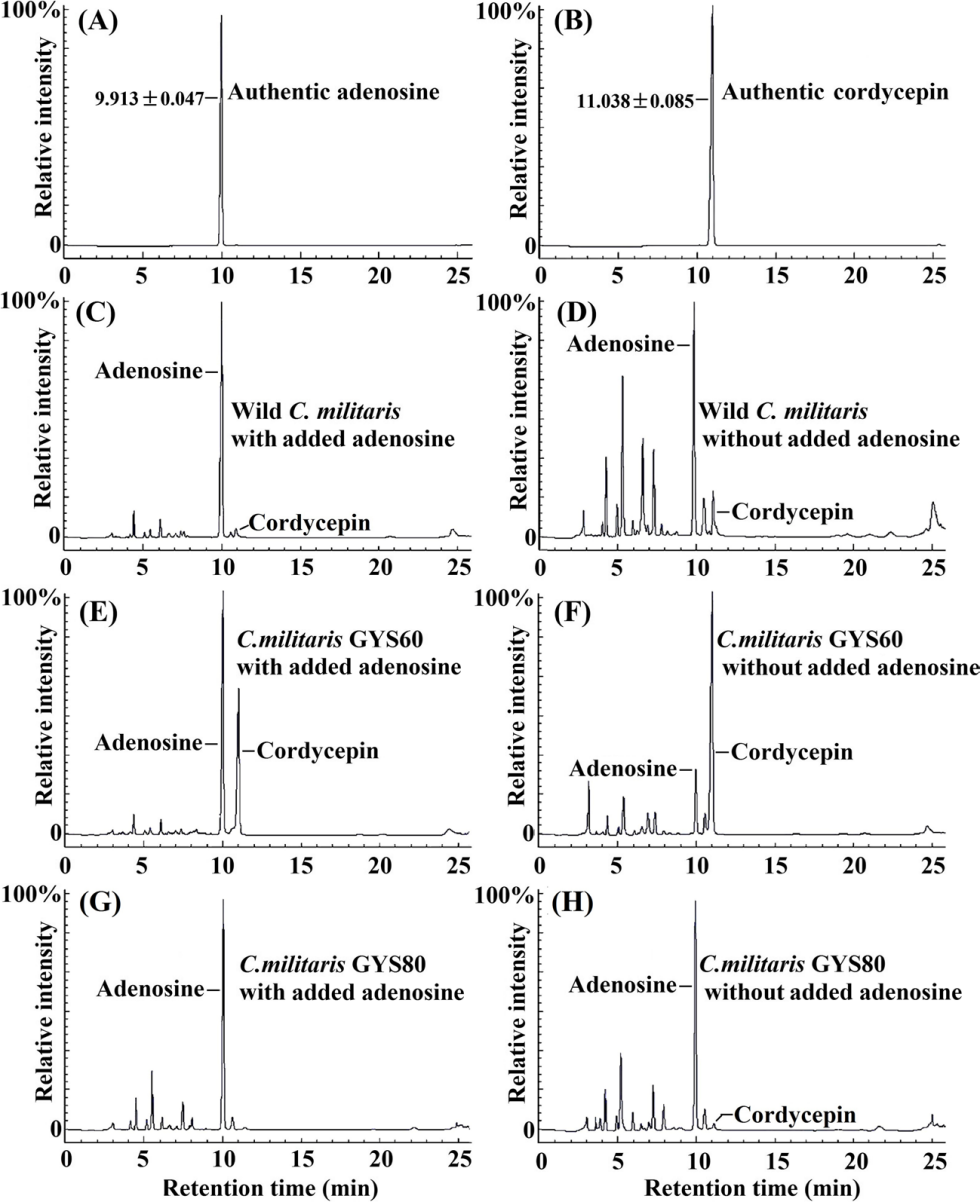
Effect of adenosine on cordycepin production. **A** HPLC profile of authentic adenosine. **B** HPLC profile of authentic cordycepin. **C** Cordycepin production by *C. militaris* with added adenosine. **D** Cordycepin production by *C. militaris* without added adenosine. **E** Cordycepin production by GYS60 with added adenosine. **F** Cordycepin production by GYS60 without added adenosine. **G** Cordycepin production by GYS80 with added adenosine. **H** Cordycepin production by GYS80 without added adenosine

Adenosine is the precursor of cordycepin biosynthesis (Kredich and Guarino 1961). If adenosine → 3′-AMP → 2′-C-3′-dA → cordycepin is the only synthesis pathway in *C. militaris*, the production of cordycepin will be greatly increased by adding adenosine during fermentation. Therefore, we investigated the effect of adenosine addition on the production of cordycepin in *C. militaris*, GYS60, and GYS80 (Fig. 5), to verify existence of a complementary cordycepin biosynthesis pathway.

At the seventh day, adenosine was added (final concentration 1.0 mg/mL) to the shaker flasks, and at the 15th day, the fermentation broths of each strain were sampled to measure the content of adenosine and cordycepin (Fig. 5, Table 4). Without added adenosine, the cordycepin yield of *C. militaris* was 0.381 ± 0.010 mg/mL, and a large amount of adenosine was produced (Fig. 5D). After we added adenosine to the *C. militaris*, the yield of cordycepin increased by 51.2% (Table 4). The amount of adenosine added was not in proportion to the increase in cordycepin yield (Fig. 5C), which confirmed that the existing main pathway of cordycepin biosynthesis did exist. In GYS60 fermentation, the cordycepin yield was 7.883 g/L without added adenosine. After adenosine was added to GYS60, the production of cordycepin increased by only 10.1% (Fig. 5E), which indicated that the main pathway of cordycepin biosynthesis had become saturated. In the presence of added adenosine, the cordycepin yield of GYS80 did not increase but decreased by 71.4% (Fig. 5G, H). These results indirectly proved that there was another pathway of cordycepin biosynthesis in GYS60, which increased production of 3′-AMP and/or 2′-C-3′-dA instead of increasing the content of adenosine and resulting in a significant increase in cordycepin production (Fig. 5F, Fig. 4).

**Table 4:** Cordycepin production and adenosine content in *C. militaris* strains with or without added adenosine (*n* = *3*, *¯x* ± *SD*)

Strains	No adenosine added			1.0 mg/mL adenosine added			
	Adenosine average ± SD (mg/mL)	Cordycepin average ± SD (mg/mL)	Cordycepin yield (%) *cf C. militaris*	Adenosine average ± SD (mg/mL)	Adenosine change (%)	Cordycepin average ± SD (mg/mL)	Cordycepin change (%)
*C. militaris*	1.514 ± 0.002	0.381 ± 0.010	control	10.010 ± 0.084	+ 561.1	0.576 ± 0.016	+ 51.2
GYS60	1.645 ± 0.068	7.883 ± 0.108	2070	12.254 ± 0.061	+ 644.9	8.673 ± 0.077	+ 10.1
GYS80	3.177 ± 0.096	0.154 ± 0.016	40	5.730 ± 0.017	+ 80.4	0.044 ± 0.001	− 71.4

After 1.0 mg/ml (final concentration) adenosine was added to the three strains, adenosine content was increased to different degrees, which indicated that the genes responsible for producing adenosine were not downregulated. Compared with *C. militaris*, the increase of cordycepin production was 10.1% in GYS60, which suggested that expression of four key genes of the main cordycepin synthesis pathway was downregulated. In GYS80, the production of cordycepin decreased the most, which indicated that the expression of the four key genes was downregulated the most. These data demonstrated that cordycepin was synthesized in an alternative metabolic route in GYS60

### Analysis of transcriptome of four known key synthetic genes of cordycepin synthesis

**Fig. 6:**
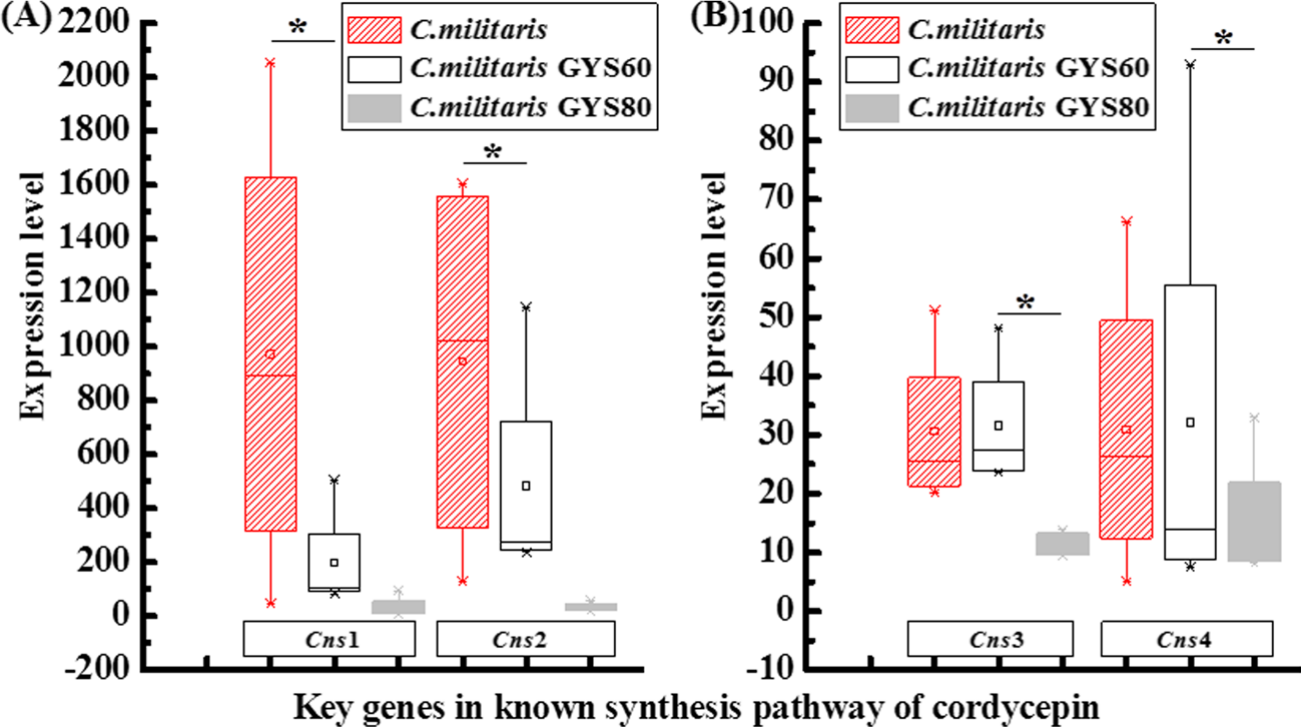
Transcriptome analysis of four key synthetic genes of cordycepin. Asterisk (*) indicates significance at *P* < 0.01 compared with control *C. militaris.* Note: *C. militaris* indicates wild type; GYS60 is high-cordycepin- producing; GYS80 is low-cordycepin-producing; *Cns1* encodes oxidoreductase/dehydrogenase; *Cns*2 encodes phosphohydrolases; *Cns*3 encodes a C-terminal phosphoribosyltransferase; *Cns*4 encodes ATP-binding cassette transporters

After transcriptome analysis of *C. militaris*, GYS60, and GYS80, we analyzed the expression levels of four known key genes, *Cns*1, *Cns*2, *Cns*3, and *Cns*4, in the main pathway of cordycepin biosynthesis to confirm the complementary cordycepin biosynthesis pathway in GYS60. The four key genes encode oxidoreductase (*Cns*1), phosphorylase (*Cns*2), phosphotransferase (*Cns*3), and channel protein (*Cns*4) (Xia et al. 2017). In *C. militaris*, the expression levels of the two most critical genes *Cns*1 and *Cns*2 in the main pathway of cordycepin synthesis were significantly higher than expression in GYS60, whereas the expression levels of *Cns*3 and *Cns*4 were low. In the main pathway of cordycepin biosynthesis (Xia et al. 2017), compared with *C. militaris*, the expression of *Cns*1, *Cns*2, *Cns*3, and *Cns*4 was not upregulated in GYS60 (Fig. 6). However, in GYS80, the expression of these genes was significantly downregulated (Fig. 6). Compared with GYS80, the expression of these genes was significantly upregulated in GYS60 (Fig. 6). These results further indicated that cordycepin was not synthesized by the main synthetic pathway in GYS60.

### RT-qPCR Validation

**Fig. 7:**
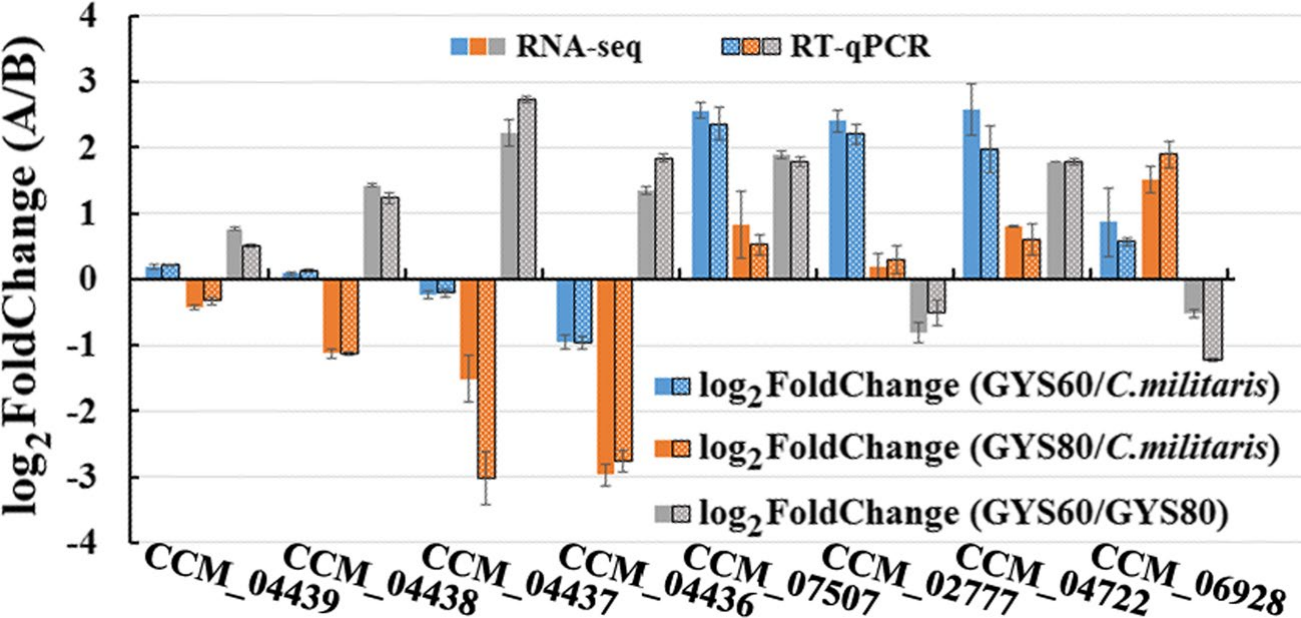
Validation of differentially expressed genes by quantitative real-time PCR. The rough bars (RT-qPCR) represent the log_2_ regulation of RT-qPCR data; the smooth bars (RNA-Seq) represent the log_2_fold change of transcriptome data. The blue, orange, and gray bars represent the log_2_ fold change (GYS60/*C. militaris*), the log_2_ fold change (GYS80/*C. militaris*), and the log_2_ fold change (GYS60/GYS80), respectively. Data are presented as the mean ± *SD* (*n* = 3)

To measure the accuracy and reproducibility of the RNA-seq data, we selected eight differentially expressed genes, CCM_07507, CCM_02777, CCM_04722, CCM_06928, CCM_04439 (*Cns*4), CCM_04438 (*Cns*3), CCM_04437 (*Cns*2), and CCM_04436 (*Cns*1), for qRT-PCR analysis. The two sets of measurements shared a similar tendency (Fig. 7). In *C. militaris*, CCM_04437 (*Cns*2) and CCM_04436 (*Cns*1) had the highest expression. In GYS60, the expression of CCM_04722, CCM_04439 (*Cns*4), and CCM_04438 (*Cns*3) was slightly higher than in *C. militaris*, whereas expression of CCM_04437 (*Cns*2) and CCM_04436 (*Cns*1) was not upregulated. In GYS60, the expression of CCM_07507, CCM_02777, CCM_04722, and CCM_06928 was the highest. These results indicated that in GYS60 the expression levels of genes CCM_07507, CCM_02777, CCM_06928 were strongly positively correlated with the biosynthesis of cordycepin, a finding that could explain the significantly increased cordycepin production by GYS60. The expression patterns of CCM_04439 (*Cns*4), CCM_04438 (*Cns*3), CCM_04437 (*Cns*2), and CCM_04436 (*Cns*1) in GYS60, related to the known main biosynthetic pathway of cordycepin, were different from expression in *C. militaris*. The validation results were consistent with the trend of RNA-seq results, which suggested that the transcriptome analysis was accurate and reliable.

## Discussion

Genes that are linked to growth are typically linked to biosynthetic/structural enzymes or they are regulatory genes. Growth is not only regulated by the mere presence of the enzymes or regulatory proteins. Growth can be stimulated by non-genome-regulated events. These are typically metabolically regulated events that can stimulate growth without necessary an up- or down-regulation of the genome. Even though there might be a downregulation of genes, a metabolic event could compensate for that gene regulation and still achieve roughly the same growth rate. Improved growth without overly affecting the growth rate itself was shown in some published papers (Vriesekoop et al. 2009, Vriesekoop and Pamment 2021). One of the other things that could affect growth rates is the fact that there are environmental conditions that can limit the growth rate regardless of how many genes are switched on.

Cordycepin production by *C. militaris* is generally conducted with one of by three culture methods, solid culture, submerged culture, and liquid surface culture (Suparmin et al. 2017). In this study, more cordycepin was produced by GYS60 than *C. militaris* or GYS80 by submerged culture. It is interesting that there were no significant differences in the growth rates between the three strains, whereas the genes related to growth were down regulated when comparing GSY60 with *C. militaris*. These phenomena suggest that the structures of some genes were destroyed by the multifunctional plasma mutagenesis system (Zhang et al. 2020), leading to changes in diversity of biological traits. The upregulation of genes related to development process, detoxification, antioxidant activity, and molecular transducer activity may compensate for the downregulation of genes related to mycelial growth (Fig. 3A), ultimately resulting in no significant difference in mycelial growth rates between the three strains (Table 2).

We compared de novo transcriptomic data of artificially cultured *C. militaris* wild-type, high-yield GYS60, and low-yield GYS80 strains to identify gene expression and function in cordycepin biosynthesis. An average of 27,194,815, 23,510,128, and 25,359,486 clean reads were obtained for GYS60, C. militaris, and GYS80, respectively. The differentially expressed genes had high similarity to the public databases COG, GO, KEGG, KOG, Pfam, Swiss Prot, eggNOG, and NR, which indicated that the transcriptome sequencing data were well assembled, and differential gene expression was well annotated.

CCM_04722 is annotated as a transferase in the Gene Ontology (GO) database (GO: 0016740), whereas, in the Clusters of Orthologous Groups (COG) of Proteins database, it is annotated as encoding a protein related to the synthesis, catalysis, and transport of secondary metabolites. The expression of CCM_04722 in GYS60 was significantly upregulated with log_2_ (FC) 1.977596036 (Table 2). Therefore, we speculated that cordycepin synthesized in GYS60 could be moved out of the cell by an ATP-binding cassette transporter encoded by *Cns4* and a transport protein encoded by CCM_04722, so that the concentration of cordycepin in the cytoplasm remained low and cordycepin synthesis could continue (Fig. 3A).

CCM_07507 is annotated as an oxidoreductase in the GO database (GO: 0016491), whereas it is a gene related to energy production and transformation in the COG database. The expression of CCM_07507 in GYS60 was significantly upregulated with log_2_ (FC) 2.364553152 (Table 2). Therefore, we speculated that the protein encoded by CCM_07507 can promote ATP synthesis in GYS60 (Fig. 3A). Under normal conditions, ATP and ADP are in equilibrium. However, in GYS60, the ATP synthesis rate is accelerated, resulting in more ATP. This increase leads to more ATP being converted by adenylate cyclase (encoded CCM_06928) into 3′,5′-cyclic AMP. Cyclic AMP is then converted by 3′,5′-cyclic AMP phosphodiesterase (encoded CCM_02777) into 3′-AMP (Fig. 4).

In GYS60, expression of eight genes was significantly upregulated (Table 2). Five of these genes were related to purine metabolism, one gene was related to ATP energy generation, one gene was related to secondary metabolite transport, and one gene was related to RNA processing and degradation. Further study is needed to determine whether the significant upregulation of these eight genes was due to the increased activity of *trans*-acting regulatory factors.

During fermentation, we added the cordycepin precursor adenosine to verify the existence of a complementary pathway of cordycepin biosynthesis in *C. militaris,* GYS60, and GYS80. In *C. militaris* and GYS60 strains, the yield of cordycepin increased by 51.2% and 10.1%, respectively, which was obviously out of proportion to the amount of added adenosine (Fig. 5, Table 3). The data indicated that the low expression of the nucleotide kinase (*Cns*3) gene in GYS60 (Table 2) led to a reduced amount of adenosine converted into 3′-AMP.

The differential gene expression of *C. militaris*, GYS60, and GYS80 was analyzed by transcriptome sequencing. In comparison with *C. militaris*, in GYS60, *Cns*1, *Cns*2, *Cns*3*,* and *Cns*4 in the main pathway of cordycepin biosynthesis were not upregulated (Table 2), which demonstrated that a large amount of cordycepin in GYS60 was achieved by the complementary pathway. The expression of *Cns*1, *Cns*2, *Cns*3, and *Cns*4 in GYS80 was significantly reduced, and the expression of the four genes in the complementary pathway was also significantly reduced (Table 2). Therefore, adenosine added during fermentation did not improve the production of cordycepin in GYS60.

## Conclusion

In the high-cordycepin-yielding strain GYS60, the ATP production rate was accelerated, which resulted in an increase in the production of 3′,5′-cyclic AMP; the elevated cyclic AMP level accelerated the accumulation of 3′-AMP. After 3′-AMP entered the last two steps of the main pathway, cordycepin continued to be synthesized. This cordycepin was transported out of the cell by two transporters, which maintained a low internal concentration of cordycepin and avoided cellular damage. Ultimately, the cordycepin yield of GYS60 was greatly increased. These findings have improved our understanding of genes involved in the biosynthesis of cordycepin and other secondary metabolites in *C. militaris*.

## Data Availability

Data generated or analyzed during this study are provided in full within the published article. The transcriptomic data are uploaded at https://www.scidb.cn/en/s/nmiqYn.
